# Antibiofilm Activity of Small-Molecule ZY-214-4 Against *Staphylococcus aureus*

**DOI:** 10.3389/fmicb.2021.618922

**Published:** 2021-02-03

**Authors:** Jingyi Yu, Lulin Rao, Lingling Zhan, Yan Zhou, Yinjuan Guo, Xiaocui Wu, Zengqiang Song, Fangyou Yu

**Affiliations:** ^1^Department of Laboratory Medicine, The First Affiliated Hospital of Wenzhou Medical University, Wenzhou, China; ^2^School of Pharmaceutical Sciences, Wenzhou Medical University, Wenzhou, China; ^3^Department of Clinical Laboratory, Shanghai Pulmonary Hospital, Tongji University School of Medicine, Shanghai, China; ^4^Shanghai Key Laboratory of Tuberculosis, Shanghai Pulmonary Hospital, Tongji University School of Medicine, Shanghai, China

**Keywords:** *Staphylococcus aureus*, small-molecule compound, biofilm, *icaA*, cell aggregation

## Abstract

*Staphylococcus aureus* is the most important pathogenic bacteria in humans. As the resistance of *S. aureus* to existing antibiotics is increasing, there is an urgent need for new anti-infective drugs. *S. aureus* biofilms cause persistent infections and resist complete eradication with antibiotic therapy. The present study investigated the inhibitory effect of the novel small-molecule ZY-214-4 (C_1__9_H_1__1_BrNO_4_) on *S. aureus* biofilm formation. At a subinhibitory concentration (4 μg/ml), ZY-214-4 had no effect on the growth of *S. aureus* strains and also showed no cytotoxicity in human normal bronchial epithelial cells (Bease-2B). The results of a semi-quantitative biofilm test showed that ZY-214-4 prevented *S. aureus* biofilm formation, which was confirmed by scanning electron microscopy and confocal laser scanning microscopy. ZY-214-4 significantly suppressed the production of polysaccharide intercellular adhesion and prevented cell aggregation, and also inhibited the mRNA expression of *icaA* and other biofilm-related genes (*eno*, *clfA/B*, *fnbB*, *fib*, *ebpS*, *psm*α, and *psm*β) in clinical *S. aureus* isolates. Thus, at a subinhibitory concentration, ZY-214-4 inhibits biofilm formation by preventing cell aggregation, highlighting its clinical potential for preventing or treating *S. aureus* infections.

## Introduction

“An antimicrobial agent” is defined as a chemical produced by microbes that can inhibit the growth of and even destroy bacteria and other microorganisms (Waksman, 1947). Most antimicrobial agents exert bacteriostatic or bactericidal effects (Gordon et al., 2013). *Staphylococcus aureus* can cause a range of infections from mild skin and soft tissue to serious life-threatening infections (Bauer and Sampathkumar, 2017) that place a significant burden on healthcare systems (Dayan et al., 2016). In recent years, virulence suppression strategies have emerged to combat *S. aureus* that prevent infection by inhibiting the action or production of virulence factors rather than targeting bacterial pathways (Cegelski et al., 2008; Escaich, 2008). However, *S. aureus* is resistant to almost all known antibiotics (Lowy, 2003). As such, there is a need for new compounds that can block the production of pathogenic factors and biofilm formation by *S. aureus* at sub-bacterial concentrations.

Biofilm formation presents a major challenge for the eradication of chronic *S. aureus* infections (Craft et al., 2019). The process of biofilm formation includes initial adhesion, proliferation, maturation, and diffusion (Boles and Horswill, 2011), with polysaccharide intercellular adhesin (PIA) providing a stable hydrated matrix that holds cells together in a three-dimensional (3D) structure (Atshan et al., 2012). PIA is also known as poly-N-acetyl glucosamine and is encoded by the intercellular adhesion gene (*ica*) (O’Gara, 2007). The recognition of Microbial Surface Components Recognize Adhesive Matrix Molecules (MSCRAMMs) determines the primary stage of *S. aureus* biofilm development, that is, the initial attachment to the host cell surface. These MSCRAMMs includes elastin binding protein (*ebpS*), laminin-(*eno*), fibronectin binding protein (*fnbA* and *fnbB*), fibrinogen binding protein (*fib*) and aggregation factor (*clfA* and *clfB*). But separation and diffusion also play important roles. Formylated phenol-soluble modulin (PSM-mec) isolated from hospital-acquired methicillin-resistant *S. aureus* was shown to promote the formation of biofilm on the surface of medical instruments that resisted penetration by antibiotics, resulting in an increased rate of nosocomial infection and chronic infections (Queck et al., 2009). PSMs are major facilitators of cell spreading (Tsompanidou et al., 2013); PSM deficiency was shown to negatively affect biofilm maturation and dissociation (Periasamy et al., 2012).

ZY-214-4 (C_1__9_H_1__0_BrNO_4_) is a small molecule that contains a chromone ring and N-phenyl-substituted maleimide. Chromone and its derivatives are present in natural products and pharmaceuticals as key scaffolds; and chromone derivatives have demonstrated antimicrobial activities against *Penicillium*, *Escherichia coli*, and *Shigella flexneri* (Verma and Pratap, 2010; Gaspar et al., 2014; Reis et al., 2017). Maleimide motifs are found in many natural products and drug candidates and exhibit a broad spectrum of biological activities including anti-tumor and anti-bacterial activities (Martinez et al., 2005; Wagner et al., 2009; Thoma et al., 2011). However, there has been no research to date on the antibacterial activity of chromone-maleimide hybrids.

In this study, we investigated the effect of a subinhibitory concentration of the small-molecule ZY-214-4 on *S. aureus* biofilm formation in order to assess the clinical potential of ZY-214-4 for preventing and treating persistent *S. aureus* infection.

## Materials and Methods

### Bacterial Strains

The *S. aureus* strains used in this study are listed in Table 1. Strains SA21, SA882, and SA923 were isolated from patients at The First Affiliated Hospital of Wenzhou Medical University. The medical records of patients and *S. aureus* isolates were obtained for research purposes with the approval of the Ethics Committee of The First Affiliated Hospital of Wenzhou Medical University; and written, informed consent was obtained from patients.

**TABLE 1:** Strains used in this study.

**Strain**	**MIC (μg/ml)**	**Source**
SA21	64	Tissue
SA882	64	Wound exudate
SA923	64	Sputum

### Synthesis of ZY-214-4

**FIGURE 1:**
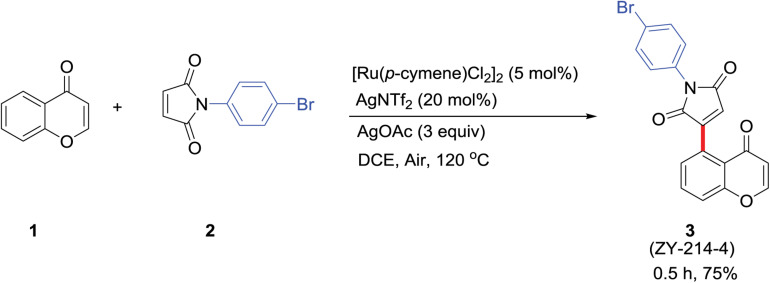
Procedure for ZY-214-4 synthesis. (1) Chromone; (2) maleimide; (3) ZY-214-4.

ZY-214-4 was synthesized at the School of Pharmacy, Wenzhou Medical University (Figure 1). Chromone 1 (0.2 mmol, 1 equiv) and maleimide 2 (0.5 mmol, 2.5 equiv) were dissolved in a 12 ml screwcapped tube with 2 ml of 1,2-dichloroethane (0.1 M). (Ru[p-cymene]Cl_2_)_2_ (0.01 mmol, 0.05 equiv), AgNTf2 (0.04 mmol, 0.2 equiv), and AgOAc (0.6 mmol,3 equiv) were added to the reaction mixture at room temperature in air, which was then heated to 120°C in a heating mantle with stirring for 0.5 h. After terminating the reaction, the reaction mixture was loaded onto a silica gel column and purified with a petroleum ether/EtOAc mixture to obtain product 3 at a yield of 75% (Supplementary Figure 2). All reagents used were of analytical grade (Zhou et al., 2020).

### Determination of Minimum Inhibitory Concentration (MIC)

ZY-214-4 was dissolved in dimethyl sulfoxide (DMSO) (Boyun, Shanghai, China) at a concentration of 20 μg/ml. The MIC of ZY-214-4 was determined with the microbroth dilution method, and was defined as the lowest concentration at which there was no visible cell growth. In order to exclude the influence of the solvent on biofilm formation, we used a DMSO control in our experiments.

### Growth Assay

*S. aureus* strains were activated twice and added to tryptic soy broth (TSB) (BD Biosciences, Franklin Lakes, NJ, United States) at 1:200 and cultured to an optical density at 600 nm (OD_600_) of 0.3. Appropriate amounts of ZY-214-4 were added to the culture to achieve final concentrations of 2, 4, and 8 μg/ml. An equivalent volume of DMSO was also tested to ensure that the vehicle did not affect cell growth, and cultures with TSB only served as the blank control. All cultures were incubated at 37°C with shaking at 220 rpm. And the OD_600_ values were measured every hour for a total of 24 h. The assay was performed in triplicate.

### Effect of ZY-214-4 on *S. aureus* Biofilm Formation

ZY-214-4 was dissolved in dimethyl sulfoxide (DMSO) to a concentration of 20 mg/ml. *S. aureus* was inoculated in 5 ml TSB and cultured at 37°C and 220 rpm. ZY-214-4 solutions were prepared at different concentrations in TSB containing 1% glucose (Kranjec et al., 2020) and was added to bacterial cultures (1:100) in 96-well plates, with 3 parallel wells at each concentration. An equivalent volume of DMSO was also tested. After incubation for 24 h, the supernatant was discarded and the wells were washed 3 times with phosphate-buffered saline (PBS). The biofilms were fixed with 99% methanol for 15 min; after discarding the supernatant, they were stained with 2% crystal violet for 10 min and rinsed under running water until the water was colorless. OD_570_ was measured after adding 70% glacial acetic acid (Kannappan et al., 2017). TSB was used as a blank control. The assay was repeated 3 times.

### Scanning Electron Microscopy (SEM) Analysis

A colony of *S. aureus* was picked from a blood agar plate and cultured overnight in 5 ml TSB medium at 37°C with shaking at 220 rpm (Kranjec et al., 2020). The culture was inoculated at 1:200 into 20 ml of TSB. A 3 ml volume of diluted bacterial solution was added to 6-well cell culture plates containing coverslips in each well and ZY-214-4 was added to achieve a final concentration 4 μg/ml, followed by incubation at 37°C for 24 h. Cultures without ZY-214-4 served as a control. The cells were cultured under the same conditions as for colony counting to ensure that the same number of bacteria were obtained. After removing the supernatant, the coverslip was washed 3 times with sterile PBS and freeze-dried for 2 h, then sputter-coated with gold for 3 min for SEM (Hitachi, Tokyo, Japan) observation.

### Confocal Laser Scanning Microscopy (CLSM)

Strains were cultured in TSB in a glass-bottomed cell culture dish (NEST Biotechnology, Wuxi, China) without shaking for 24 h (Kannappan et al., 2017), in the presence of 4 μg/ml ZY-214-4; untreated samples served as a positive control. After rinsing with 0.85% saline, the dish was air-dried and 300 μl of SYTO-9 [0.02%; component A of the LIVE/DEAD BacLight Bacterial Viability kit (Thermo Fisher Scientific, Waltham, MA, United States)] and propidium iodide (0.067%) were added for 30 min in the dark at room temperature. Optical sections were scanned by CLSM (Nikon, Tokyo, Japan) using a 60 × oil-immersion objective lens. 3D reconstruction of the images was performed using NIS-Elements software (Nikon). The assay was performed in triplicate.

### Cell Aggregation Assay

Cell aggregation was analyzed as previously described (Feldman et al., 2018). Briefly, five strains of *S. aureus* were added to 2 ml TSB at 1:200 and ZY-214-4 was added at a final concentration 4 μg/ml. The cells were cultured at 37°C and 250 rpm for 24 h; untreated samples served as the control. The cells were collected by centrifugation at 16,600×*g* for 2 min, washed 3 times with PBS, and resuspended in 3 ml PBS; OD_595_ was adjusted to 1.5 (initial OD) in a clean glass tube, and the sample was allowed to stand at room temperature for 24 h. The supernatant was removed by aspiration and the cell pellet was resuspended in 3 ml PBS. OD_595_ (final OD) was measured and the percentage of cell aggregation was determined as (final OD/initial OD) × 100%. The relative aggregation of ZY-214-4–treated samples is expressed as a percentage of the value for untreated controls (100%). The assay was performed in triplicate.

### Enzyme-Linked Dot Immunoblot Assay for PIA

*S. aureus* was inoculated on a blood agar plate and cultured at 37°C for 14–18 h. The culture was diluted to 10^7^ CFU/ml and added to a 6-well plate (2 ml per well) (Schlag et al., 2007). ZY-214-4 was then added to a final concentration 4 μg/ml, followed by incubation at 37°C for 24 h. Bacterial solution without ZY-214-4 served as a control. The culture medium was removed and 3 ml of EDTA was added to each well for resuspension of the biofilm attached to the bottom of the plate. The cells were transferred to a 1.5 ml centrifuge tube and incubated at 100°C for 5 min. After centrifugation at 8,000 rpm for 1 min, the supernatant was removed and 10 μl of 20 mg/ml proteinase K was added to 40 μl of supernatant, followed by incubation at 37°C for 2 h. A polyvinylidene difluoride membrane cut to an appropriate size was immersed in methanol for 3 min and deionized water for 15 min to activate functional groups on the membrane. A 10 μl volume of the extracted PIA sample was spotted onto the membrane, which was kept moist during the procedure. After the membrane had completely dried it was transferred to blocking solution [PBS with 0.1% Triton X100 (PBST) containing 3.5% bovine serum albumin] and incubated overnight at 4°C. The membrane was transferred to a Petri dish containing wheat germ agglutinin-conjugated horseradish peroxidase and incubated at 37°C for 1 h, then washed twice with PBST for 12 min each time followed by PBS before visualizing the signal by enhanced chemiluminescence (Affinity Bio, San Francisco, United States).

### Real-Time (RT)-PCR

*S. aureus* strains were cultured in TSB containing ZY-214-4 at an inhibitory concentration (4 μg/ml). After 12 h, total RNA was extracted from the cells and cDNA was synthesized using an RNA PCR kit (Takara Bio, Otsu, Japan). PCR was performed in a 20 μl reaction volume using Luna Universal qPCR Master Mix (New England Biolabs, Ipswich, MA, United States). The primer pairs used for RT-PCR are shown in Table 2. The assay was performed in triplicate.

**TABLE 2:** Primers used for real-time RT-PCR.

**Primer name**	**Sequence (5′–3′)**
*gyrb*-RT-F	ACATTACAGCAGCGTATTAG
*gyrb*-RT-R	CTCATAGTGATAGGAGTCTTCT
*icaA*-RT-F	GTTGGTATCCGACAGTATA
*icaA*-RT-R	CACCTTTCTTACGTTTTAATG
*psm*α-RT-F	ATGGAATTCGTAGCAAAATTATTC
*psm*α-RT-R	TAGTTGTTACCTAAAAATTTACC
*psm*β-RT-F	CCTAGTAAACCCACACCG
*psm*β-RT-R	GCTGCACAACAACATGATA
*clfA*-RT-F	CAGCGATTCAGAATCAGA
*clfA*-RT-R	GGCGGAACTACATTATTG
*clfB*-RT-F	CTGAGTCACTGTCTGAATC
*clfB*-RT-R	CTCAGACAGCGATTCAGA
*fnbB*-RT-F	GCGAAGTTTCTACTTTTG
*fnbB*-RT-R	CAACCATCACAATCAACA
*eno*-RT-F	CTCCAATTGCATTCCAAG
*eno*-RT-R	GCATCTTCAGTACCTTCA
*ebpS*-RT-F	GTGTGATGATTCGACTTG
*ebpS*-RT-R	CAGGATACAATAGAGAATACG
*fib*-RT-F	GCTGTAAACTTGTTCAAAC
*fib*-RT-R	CTGTGTTGGAAATGAATTAAG

### Cytotoxicity Analysis

The cytotoxicity of ZY-214-4 was evaluated with the Cell Counting Kit (CCK)-8 assay using Bease-2B human normal bronchial epithelial cells. The cells were seeded in a 96-well plate containing Dulbecco’s Modified Eagle’s Medium (DMEM)/F12 at 4,000 cells/well, and 10% fetal bovine serum (FBS) was added to each well, followed by incubation at 37°C and 5% CO_2_ in a humid environment for 12 h. The supernatant was discarded and the wells were washed 3 times with PBS. ZY-214-4 solution (2–8 μg/ml) was added for 24 h, with untreated cells serving as a control. The supernatant was discarded and cells were washed 3 times with PBS; DMEM/12 was added to each well along with CCK-8 reagent, followed by incubation for 1.5 h at 37°C and measurement of OD_450_. The assay was repeated 5 times. Relative cell viability (%) was determined by comparing the OD_450_ value with that of the control well containing only cell culture medium.

### Statistical Analysis

Prism 6 software (GraphPad, La Jolla, CA, United States) was used to analyze experimental data. Growth curves were analyzed with the *t*-test and one-way analysis of variance was used for all other data. A *p* < 0.05 was considered statistically significant.

## Results

### ZY-214-4 Suppresses *S. aureus* Growth

**FIGURE 2:**
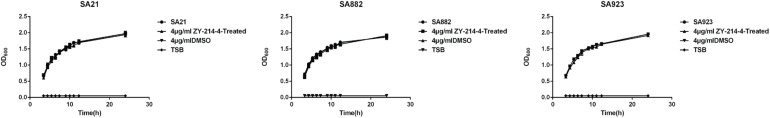
Growth curves of *S. aureus* strains cultured with ZY-214-4. TSB served as a blank control, and growth in the presence of DMSO was evaluated to eliminate the possibility that inhibition of biofilm formation is due to suppression of cell growth.

The MIC of ZY-214-4 for *S. aureus* strains SA21, SA882, and SA923 was 64 μg/ml. We generated growth curves for cells treated with various concentrations of ZY-214-4 and found that at the subinhibitory concentration of 4 μg/ml, the number of cells in the late logarithmic growth phase was consistent across strains (Figure 2). Therefore, 4 μg/ml ZY-214-4 was used for subsequent experiments.

### ZY-214-4 Prevents *S. aureus* Biofilm Formation

**FIGURE 3:**
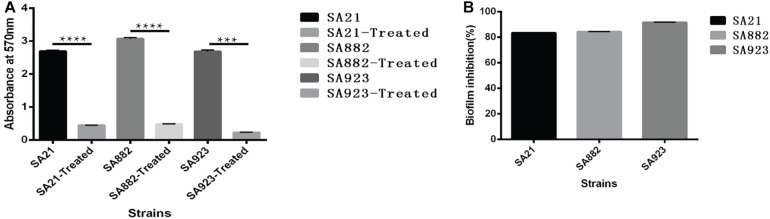
Effect of subinhibitory ZY-214-4 concentration (4 μg/ml) on *S. aureus* biofilm formation. **(A)** OD_570_ of each strain differed significantly from that of the control group (grown in the absence of ZY-214-4). **(B)** Biofilm inhibition rate of 3 *S. aureus* strains. *P < 0.05.

**FIGURE 4:**
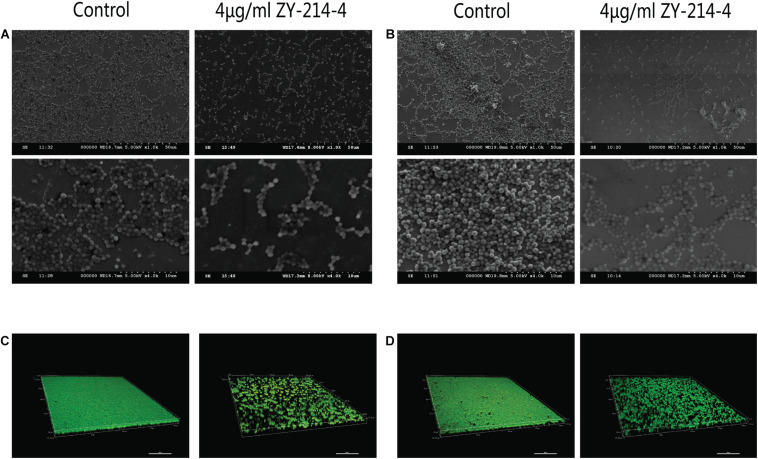
Effect of ZY-214-4 on *S. aureus* biofilm formation evaluated by microscopy. **(A–D)** The antibiofilm potential of ZY-214-4 against *S. aureus* clinical isolates SA21 **(A,C)** and SA923 **(B,D)** was visualized by SEM **(A,B)** and CLSM **(C,D)**.

*S. aureus* forms biofilm, which protects it against the action of anti-bacterial drugs. We used a semi-quantitative method to evaluate the effect of ZY-214-4 on *S. aureus* biofilm formation. *S. aureus* treated with ZY214-4 (4 μg/ml) had a 7.5–13 times lower OD_570_ than untreated cells (0.2–0.4 vs. 2.6–3.0), with biofilm formation reduced by 83.3–91.5% (Figure 3). SEM analysis of *S. aureus* biofilms showed a high degree of aggregation of SA21 and SA923 in the absence of treatment. However, in ZY-214-4–treated samples, cell aggregates covered a smaller area and were scattered, with greater distance between colonies (Figures 4A,B). Examination of 3D biofilm morphology by CLSM showed that compared to the control sample, the thickness of the biofilm was decreased and the area covered by the biofilm was reduced by ZY-214-4 treatment (Figures 4C,D).

### ZY-214-4 Prevents *S. aureus* Aggregation

**FIGURE 5:**
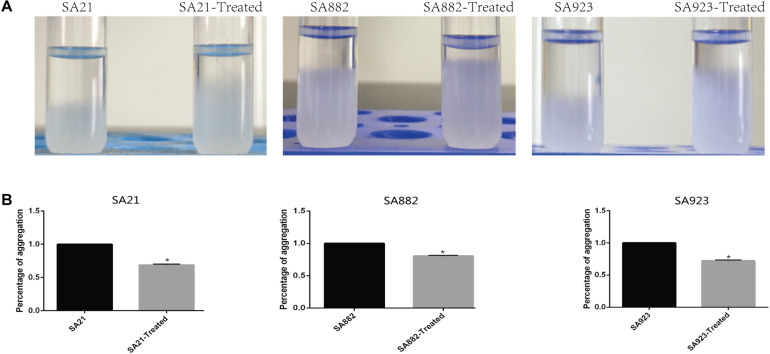
Effect of ZY-214-4 on *S. aureus* cell aggregation. **(A)** Micrographs of *S. aureus* autoaggregation upon ZY-214-4 treatment. **(B)** Quantitative analysis of *S. aureus* aggregation. ZY-214-4 inhibited aggregation relative to untreated control cells. *P < 0.05.

Cell aggregation plays an important role in biofilm formation. Autoaggregation was observed after incubating *S. aureus* strains for 24 h under static conditions (Figure 5A). The cell aggregation rate was significantly decreased by treatment with ZY-214-4 to 68.81–83.14% of the value in untreated control samples (Figure 5B).

### ZY-214-4 Inhibits PIA Production by *S. aureus*

**FIGURE 6:**
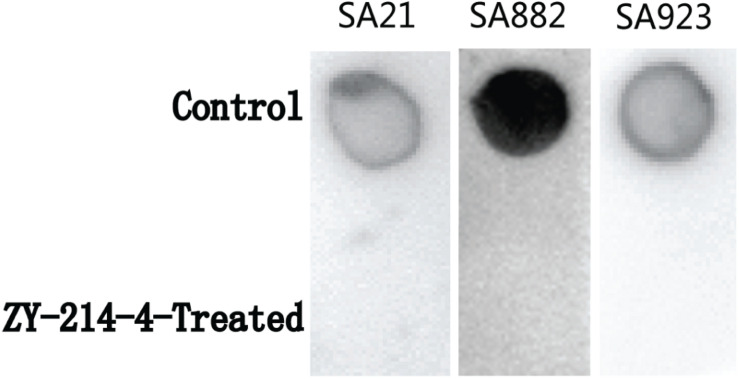
Effect of ZY-214-4 (4 μg/ml) on *S. aureus* PIA production.

Extracellular PIA is the main component of staphylococcal biofilms. To investigate the effect of ZY-214-4 on the production of biofilm matrix, we measured PIA release in cultures. As shown in the Figure 6, when the PIA antigen was diluted to 5,000-fold, as determined by the semi-quantitative determination of the WGA-HRP conjugate using the dot blot 96 system, among the strains treated with ZY-214-4 and untreated strains. This indicates that ZY-214-4 prevented *S. aureus* biofilm formation by inhibiting the production of PIA.

### ZY-214-4 Suppresses the Expression of *S. aureus* Biofilm-Related Genes

**FIGURE 7:**
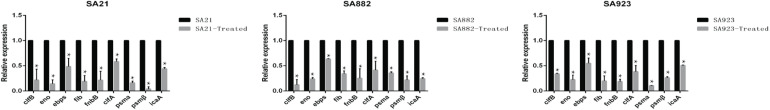
Effect of ZY-214-4 on relative expression of biofilm-related genes in *S. aureus*. Values represent mean ± *SD*. Cells grown without ZY-214-4 served as a control. *P < 0.05.

PIA synthesis depends on the expression of the *icaA* gene, but genes encoding microbial surface components that recognize MSCRAMM also play a key role in biofilm formation. PSMs influence bacterial cell aggregation, and their absence can affect biofilm maturation and dissociation. To clarify the mechanism by which ZY-214-4 blocks *S. aureus* biofilm formation, we compared the expression of biofilm-related genes between untreated and ZY-214-4-treated *S. aureus* by RT-PCR. The results showed that the expression of *icaA*, *eno*, *clfA/B*, *fnbB*, *fib*, *ebpS*, *psm*α, and *psm*β genes was downregulated to varying degrees in the latter cells (Figure 7).

### ZY-214-4 Is Nontoxic to Bease-2b Cells

**FIGURE 8:**
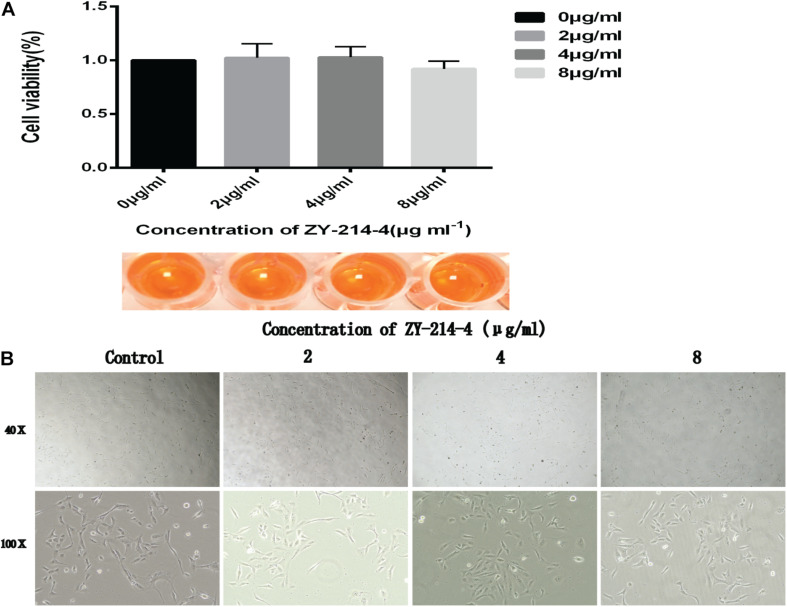
Effect of ZY-214-4 on the growth and morphology of Bease-2B human normal bronchial epithelial cells. **(A)** CCK-8 assay. **(B)** Cell morphology and number evaluated by light microscopy.

We evaluated the cytotoxicity of ZY-214-4 with the CCK-8 assay using Bease-2B human normal bronchial epithelial cells. There was no difference in viability between control and ZY-214-4 treated groups (Figure 8A) and microscopic observation revealed no differences in cell morphology (Figure 8B), indicating that ZY-214-4 is not cytotoxic at a subinhibitory concentration.

## Discussion

Biofilms are fixed communities of bacterial cells attached to each other and embedded in a self-produced extracellular polymer (EPS) matrix (Flemming and Wingender, 2010). Biofilms can be formed on various surfaces including ducts, implants, prosthetics, and implanted medical devices (Costerton et al., 1999). There is evidence that cells in biofilms on biotic or abiotic surfaces are 1,000 times more resistant to conventional drugs than planktonic cells (Parsek and Singh, 2003; Verderosa et al., 2019). Once formed, a biofilm is difficult to eliminate and can develop into a chronic and persistent infection.

In this study, we synthesized the small-molecule compound ZY-214-4 and evaluated its antibacterial efficacy against clinical isolates of *S. aureus* obtained from different infection sites. A high concentration of ZY-214-4 inhibited the growth of *S. aureus* (Supplementary Figure S1) while no effect was observed at the subinhibitory concentration of 4 μg/ml. We therefore used the latter in our experiments in order to exclude the possibility that ZY-214-4 prevents *S. aureus* biofilm formation by suppressing cell proliferation.

Downregulation of the *ica* gene encoding PIA is a known mechanism of *S. aureus* biofilm inhibition (Cramton et al., 1999). We found that PIA production by *S. aureus* was decreased by treatment with 4 μg/ml ZY-214-4. This is consistent with the relevant research results of Lee et al. (2013, 2014) on compounds inhibiting the formation of bacterial biofilms. Bacterial cell aggregation is an initial step in biofilm formation (Arciola et al., 2012). In staphylococci, PIA promotes cell–cell adhesion, which is important for cell aggregation following attachment to a surface (Mack et al., 1994; Heilmann et al., 1996). PSMs—especially PSMβ—play an important role in cell aggregation (Periasamy et al., 2012; Kim et al., 2020). In this study we found that downregulation of the *psm* gene in the presence of ZY-214-4 was associated with a reduction in *S. aureus* cell aggregation. A consistent conclusion can be found in the relevant research of Lee et al. (2016). Additionally, the expression of genes involved in the initial attachment of cells to each other (*eno*, *clfA/B*, *fnbB*, *fib*, and *ebpS*) was also decreased. Thus, ZY-214-4 prevents biofilm formation by *S. aureus* by blocking the production of PIA and cell aggregation.

Importantly, ZY-214-4 had no cytotoxicity in Bease-2B epithelial cells at a subinhibitory concentration, suggesting that it is safe for use in humans.

This study had some limitations. For example, the mechanism by which ZY-214-4 regulates the expression of *icaA*, *psm*, and other biofilm-related genes remains to be determined. Nonetheless, we demonstrated that the small-molecule ZY-214-4 is non-toxic to human cells and effective in preventing biofilm formation by *S. aureus* at a subinhibitory concentration. These findings highlight the clinical potential of ZY-214-4 for the prevention and treatment of chronic *S. aureus* infections.

## Data Availability Statement

The raw data supporting the conclusions of this article will be made available by the authors, without undue reservation.

## Ethics Statement

Written informed consent was obtained from the individual(s) for the publication of any potentially identifiable images or data included in this article.

## Author Contributions

JY, LR, and YZ designed the work and analyzed and interpreted the data for the work. JY and YG drafted the work and revised it critically for important intellectual content. FY provided approval for publication of the content. LZ, ZS, and XW participated in the experimental design and data analysis. FY agreed to be accountable for all aspects of the work in ensuring that questions related to the accuracy or integrity of any part of the work are appropriately investigated and resolved. All authors read and approved the final manuscript.

## Conflict of Interest

The authors declare that the research was conducted in the absence of any commercial or financial relationships that could be construed as a potential conflict of interest.

## Supplementary Material

Supplementary Figure 1 Growth curves of *S. aureus* strains cultured with ZY-214-4 (>4 μg/ml).

Supplementary Figure 2 NMR identification of ZY-214-4.
