# Supplementary figures and images for: Antibiofilm Activity of Small-Molecule ZY-214-4 Against Staphylococcus aureus
Source: Front Microbiol. 2021 Feb 3;12:618922. doi: 10.3389/fmicb.2021.618922 (PMC7886693; doi:10.3389/fmicb.2021.618922)

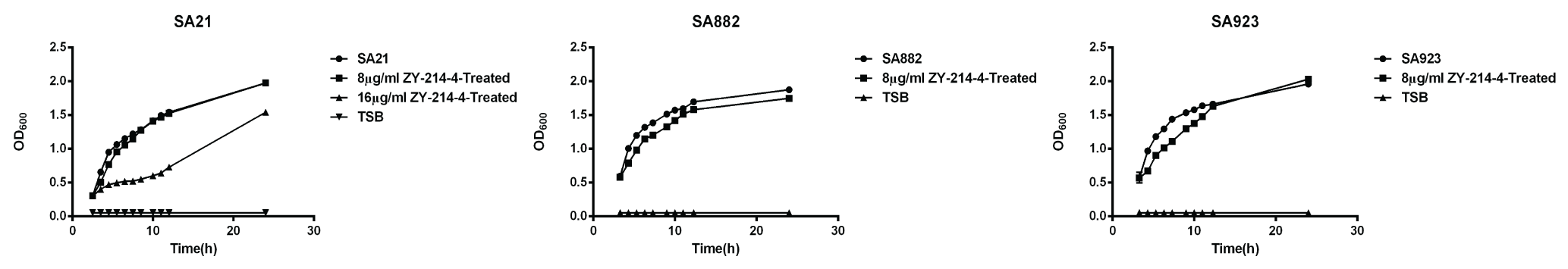

Supplement: Supplementary Figure 1 — Growth curves of S. aureus strains cultured with ZY-214-4 (>4 μg/ml). [file Image_1.TIF]

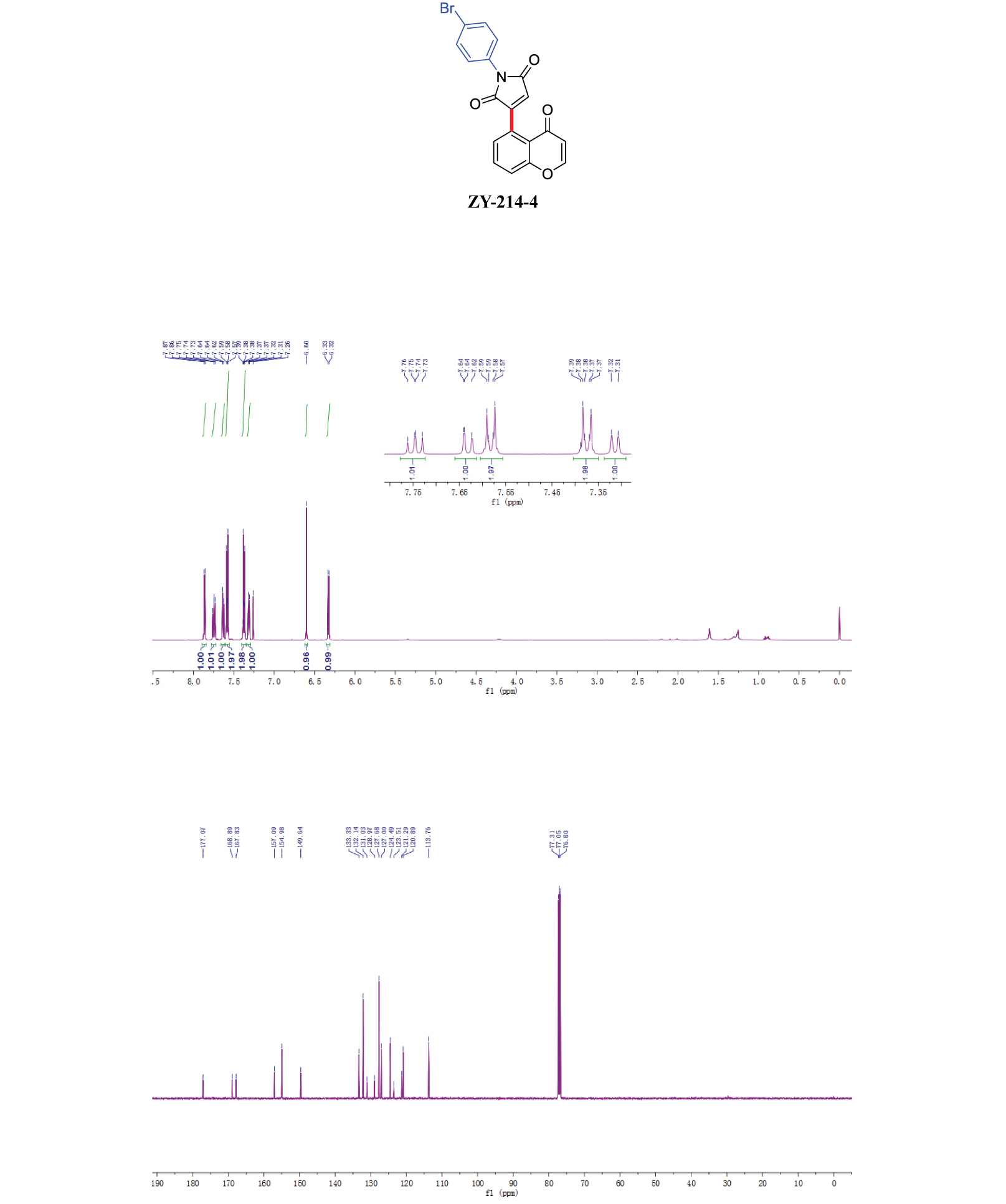

Supplement: Supplementary Figure 2 — NMR identification of ZY-214-4. [file Image_2.TIF]
